# Detection and genetic characterisation of porcine circovirus 3 from pigs in Sweden

**DOI:** 10.1007/s11262-018-1553-4

**Published:** 2018-03-21

**Authors:** Xingyu Ye, Mikael Berg, Caroline Fossum, Per Wallgren, Anne-Lie Blomström

**Affiliations:** 1 Guangyuan Center for Animal Disease Control and Prevention, Guangyuan, 628017 China; 2 Department of Biomedical Sciences and Veterinary Public Health, Swedish University of Agricultural Sciences, Box 7028, 750 07 Uppsala, Sweden; 3 National veterinary institute (SVA), 751 89 Uppsala, Sweden

**Keywords:** Genetic characterisation, Porcine circovirus 3, Sweden, Mini PCV-like virus

## Abstract

Porcine circovirus 3 (PCV3) is a newly detected circovirus belonging to the family *Circoviridae* with a circular ssDNA genome of 2000 bp that encodes two proteins—the replicase protein and the capsid protein. PCV3 was discovered for the first time in the US in 2016. After this initial discovery, PCV3 was detected in other parts of the world such as in China, South Korea, Italy and Poland. In this study, 49 tissue samples from Swedish pig herds were screened for PCV3 using PCR and 10 samples were positive and one was uncertain. The entire PCV3 genome and a mini PCV-like virus (MPCLV) were obtained from one of these samples. These two viruses showed a high sequence identity to PCV3 viruses from other countries as well as to MPCLV from the US. However, the sequence identity to PCV1 and 2 was only 31–48% on amino acid level. This is the first detection and complete genetic characterisation of PCV3 in Swedish pigs. It is also interesting to note that one of the positive samples was collected in 1993, showing that PCV3 has been present for a long time.

Circoviruses are small non-enveloped, icosahedral ssDNA viruses belonging to the genus *circovirus* in the family *Circoviridae* [1]. Globally, there are three different types of characterised Porcine Circoviruses (PCV) identified in pig herds so far—PCV1, 2 and 3. PCV1 is widely acknowledged to be non-pathogenic [2], whereas PCV2 is associated with a series of porcine circovirus-associated diseases, which include a broad spectrum of clinical symptoms generating worldwide economic losses in pig production [3]. PCV3 was first identified from piglets with clinical disease of anorexia, weight loss and swollen joints in US in 2016 [4]. After this discovery, PCV3 has been detected in other countries including, China, South Korea, Poland and Italy [5–9]. PCV3 has a genome of 2000 nucleotides and shows a low amino acid identity to PCV2 (26% capsid; 48% replicase) [4]. However, the replicase (Rep) gene had 95% amino acid identity to the mini PCV-like virus (MPCLV) strain SFBeef and to SFporkNMW2 [10, 11]. As there is no report on the existence of PCV3 in Scandinavia, this study aimed to detect and genetically characterise PCV3 in Sweden.

DNA from 49 archived lymph node samples collected from 1993 to 2007, which have previously been investigated for PCV2, Torque teno sus virus (TTSuV) and Porcine Bocavirus (PBoV) [12], was used. Two pairs of primer (Rep-F 5′-TTTACGATAAACAACTGGACCC-3′, Rep-R 5′-CATCTTCTCCGCAACTTCAG-3′, Cap-F 5′-CGTAGAAGTCTGTCATTCCA-3′ and Cap-R 5′-AAGACGACCCTTATGCGG-3′) were designed, based on the PCV3-US/SD2016 (KX966193), to detect the Rep gene and capsid (Cap) gene, respectively. The PCR of the 49 DNA templates was run according to the following protocol: 1 × PCR buffer, 1.5 mM MgCl_2_, 0.2 mM dNTP, 0.2 mM of each primer and 0.625U AmpliTaq Gold DNA polymerase (Thermo Fisher). Amplification was initiated with a 10 min heating step at 95 °C followed by 35 cycles of 15 s at 95 °C, 30 s at 55 °C and 35 s at 72 °C. The amplification was stopped with a final extension step at 72 °C for 5 min. The PCR products of expected length were purified with Thermo Fisher PCR purification kit according to the manufacturer’s instructions and sent for sequencing at Macrogen. In total, 10 out of 49 samples were positive for PCV3 and one was uncertain (only positive in the Rep PCR). Out of the 10 positive samples, nine samples came from healthy pigs while one of the pigs came from a post-weaning multisystemic wasting syndrome (PMWS) positive herd.

Since the amount of DNA in a single positive sample was limited, we designed a PCV3-specific rolling circle amplification (PCV3-RCA) in order to increase the DNA load of PCV3. The PCV3-RCA contained a mix of 8 exo-resistant PCV3-specific primers (P1 5′-AGGTCTGTAGG-3′, P2 5′-ACTCGTAGCA-3′, P3 5′-GGAAATCTGAC-3′, P4 5′-ACCACTCCTC-3′, P5 5′-GTTTACCTGTG-3′, P6 5′-AAATCTGACCC-3′, P7 5′-GTAACG AATCC-3′, P8 5′-AATACTCCACC-3′) at a final concentration of 0.4 mM each, 1 × phi29DNA polymerase buffer, 0.1 μg bovine serum albumin, 0.67 mM dNTP, 8U phi29 DNA polymerase (Thermo Fisher) as well as 2 μl of denatured DNA. Four primers (Rep-F1 5′-CGAGAATTCCGAGATTGGCGAAGATTCC-3′, Rep-R1 5′-CGAGAATTCTCTCGAGGTAATCCCCCTCT-3′, Cap-F1 5′-CGAGAATTCGCATAAGGGTCGTCTTGGAG-3′, Cap-R1 5′-CGAGAATTCTATGCGGAAAGTTCCACTCG-3′) were designed to amplify the entire genome. The amplification was performed using 2 μl of 10 times diluted RCA products as templates and the purified positive PCR products were inserted into the pJET vector (Thermo Fisher) and sent for sequencing at Macrogen. SeqMan (Lasergene 9, DNASTAR) was used to assemble and edit the sequences. One PCV3 genome sequence (PCV3/SWE84/2004) and one mini PCV3-like virus (MPCLV/SWE84/2004) genome sequence were obtained from a sample collected in 2004. The genome sequences were deposited into the GenBank database under the accession numbers MG765473 and MG765474, respectively. Like the previously reported PCV3 sequences, PCV3/SWE84/2004 was 2000 nucleotides in length including two open reading frames (ORF) which encode the 296-aa Rep protein and the 214-aa Cap protein. MPCLV/SWE84/2004 which was 839 nucleotides in length contained one ORF encoding a putative Rep protein and two ORFs of hypothetical protein of unknown function but no Cap protein was identified.

The conserved stem-loop motif (TAGTATTAC) which initiates the replication of other circoviruses was found in the 5′ untranslated region (UTR) of PCV3/SWE/2004 and MPCLV/SWE84/2004 using MEME Suite 4.12.0 [13]. Three rolling circle replication motifs (I [FTINN], II [PHLQG] and III [YCKK]), three helicase domains (Walker A [GKEVGKS], Walker B [ILDDF] and Walker C [ITSN]) and three motifs ([WWDGY], [DDFYGWVP] and [DRYP]) with unclear function were identified in the Rep sequence of PCV3/SWE84/2004 while MPCLV/SWE84/2004 only possessed Motif I, II, III and Walker A [5, 13]. Protein sequence identity analysis was performed and a neighbour-joining phylogenetic tree with a bootstrap value of 1000 was constructed using Mega5 [14].

The protein sequence identity results are shown in Table 1. Across all proteins, both PCV3/SWE84/2004 and MPCLV/SWE84/2004 displayed highest identity to the PCV3 strain Guangdong-HY1/2016 (100%) from China (GenBank accession number MF589102) and 99–100% identity to available PCV3 sequences from other countries. Compared to the Circoviridae SFBeef and PorkNW2, identified in beef and pork in US in 2014 [11], with no identified Cap gene, a high sequence identity (96–99%) was displayed. For PCV1 and 2, the sequence identity ranged from 24 to 44%. The similarities were not uniform across the entire genome sequence and the Rep protein was more similar than the Cap protein. Moreover, the phylogenetic tree (Fig. 1) showed that PCV3/SWE84/2004 and MPCLV/SWE84/2004 clustered with the PCV3 isolates from Guangdong Province, China and US, and were relatively distant from PCV1 and PCV2.

**Table 1:** Protein sequence identity in percentage

	PCV3/US	PCV3/China	PCV3/S Korea	PCV3/Italy	PCV3/Brazil	SFBeef	PorkNW2	PCV2	PCV1
	KX778720	MF589102	MF611876	MF162298	MF079253	KM111537	HQ738638	AY754017	AF071879
PCV3/SWE/84-REP MG765473	99	100	100	99	99	99	96	44	43
PCV3/SWE/84-CAP MG765473	100	100	99	99	99	–	–	27	24
MPCLV/SWE/84 MG765474	99	100	100	99	100	99	97	42	42

The complete amino acid sequences of each protein are compared between the Swedish sequences and those of representatives from different countries. If no comparison was possible, a “–” was added in the table

**Fig. 1:**
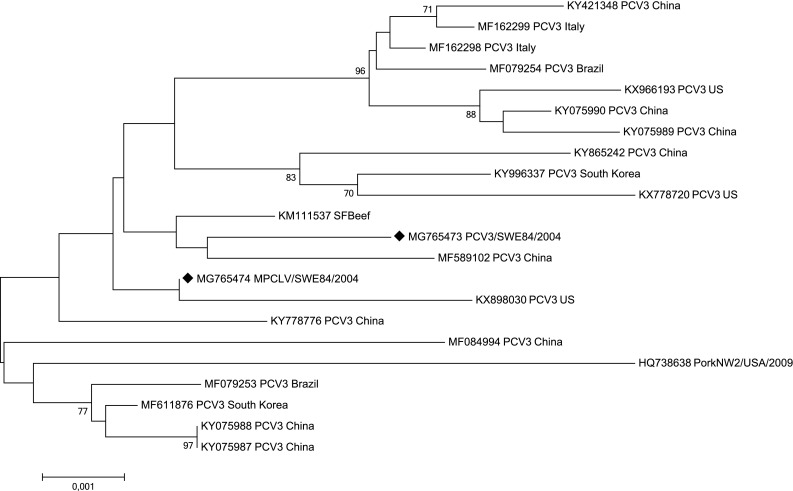
Phylogenetic relationship between porcine circoviruses. The evolutionary history was inferred using the Neighbour-joining method, with a bootstrap test of 1000 replicates. The evolutionary distances were computed using the *p* distance method. The complete genomic sequence was used and the viral sequences characterised in the present study are marked with diamonds

In previous studies, PCV3 has been detected in pigs with cases of cardiac and multisystemic inflammation [4], porcine dermatitis and nephropathy syndrome, reproductive failure, aborted foetuses [15] and pyrexia and respiratory disease [9, 16, 17]. Meanwhile, PCV3 co-infections with PCV2, TTSuV, porcine hemagglutinating encephalomyelitis virus and porcine reproductive and respiratory syndrome virus have also been reported [4, 15, 16]. The co-infection rate of PCV3 with PCV2 was 28.3% in aborted foetuses and respiratory diseased piglets [17]. In this study, 10 samples including one from a PWMS-affected herd were positive for PCV3 and the detection rate (20.4%) was considerably lower than that for TTSuV1 (73.5%), TTSuV2 (87.8%) and PBoV (67.3%) using the same sample set. 70% of the PCV3 positive pigs were coinfected with TTSuV1 and TTVSuV2, respectively, and 50% were coinfected with PBoV. In addition, an entire PCV3-associated viral genome which was tentatively named MPCLV/SWE84/2004 was identified and genetically characterised. As is seen for Circoviridae SFBeef, the MPCLV possessed a Rep protein. Similar to PCV2-like truncated and rearranged genomes [18–20], the MPCLV is possibly generated when the PCV3 is passaged at high multiplicity of infection. This could result in defective interfering viral particles that only retains the essential cis-elements required for genome replication and requires a helper virus [21].

To conclude, this study is the first demonstration of the presence of PCV3 in domestic pigs in Scandinavia and the fact that the PCV3 positive samples were collected in a period spanning from 1993 to 2007 shows that the virus has been circulating in Sweden for a long time. Overall, the genetic homology between viruses from different continents seems to be very high. As other “new” viruses like PBoV, PCV3 and MPCLV are identified in pigs, it is important to start understanding the potential role that these co-infections may have on the pigs. For this, establishment of in vitro and in vivo experimental systems combined with clinical pathology is crucial.
